# Circulating proteins as potential biomarkers of sunitinib and interferon-α efficacy in treatment-naïve patients with metastatic renal cell carcinoma

**DOI:** 10.1007/s00280-013-2333-4

**Published:** 2013-11-13

**Authors:** Charles S. Harmon, Samuel E. DePrimo, Robert A. Figlin, Gary R. Hudes, Thomas E. Hutson, M. Dror Michaelson, Sylvie Négrier, Sindy T. Kim, Xin Huang, J. Andrew Williams, Tim Eisen, Robert J. Motzer

**Affiliations:** 1 Pfizer Oncology, 10646 Science Center Drive, La Jolla, San Diego, CA 92121 USA; 2 Samuel Oschin Comprehensive Cancer Institute, Cedars-Sinai Medical Center, Los Angeles, CA USA; 3 Fox Chase Cancer Center, Philadelphia, PA USA; 4 Baylor Sammons Cancer Center-Texas Oncology, P.A., Dallas, TX USA; 5 Massachusetts General Hospital Cancer Center, Boston, MA USA; 6 Centre Léon Bérard, Lyon, France; 7 Cambridge University Health Partners, Addenbrooke’s Hospital, Cambridge, UK; 8 Memorial Sloan-Kettering Cancer Center, New York, NY USA; 9 Present Address: Independent Consultant, San Diego, CA USA; 10 Present Address: Janssen Research and Development, San Diego, CA USA

**Keywords:** Metastatic renal cell carcinoma, Sunitinib, Biomarkers, Phase III clinical trial

## Abstract

**Purpose:**

We investigated potential biomarkers of efficacy in a phase III trial of sunitinib versus interferon-alpha (IFN-α), first-line in metastatic renal cell carcinoma (mRCC), by analyzing plasma levels of vascular endothelial growth factor (VEGF)-A, VEGF-C, soluble VEGF receptor-3 (sVEGFR-3) and interleukin (IL)-8.

**Methods:**

Seven hundred and fifty mRCC patients were randomized to oral sunitinib 50 mg/day in repeated cycles of a 4-week on/2-week off schedule or IFN-α 9 million units subcutaneously thrice weekly. Plasma samples collected from a subset of 63 patients on days 1 and 28 of cycles 1–4 and at end of treatment were analyzed by ELISA.

**Results:**

Baseline characteristics of biomarker-evaluated patients in sunitinib (*N* = 33) and IFN-α (*N* = 30) arms were comparable to their respective intent-to-treat populations. By univariate Cox regression analysis, low baseline soluble protein levels were associated with lower risk of progression/death (all *P* < 0.05): in both treatment arms, baseline VEGF-A and IL-8 were associated with overall survival (OS) and baseline VEGF-C with progression-free survival (PFS); in the sunitinib arm, baseline VEGF-A was associated with PFS and baseline sVEGFR-3 with PFS and OS; in the IFN-α arm, baseline IL-8 was associated with PFS. In multivariate analysis, baseline sVEGFR-3 and IL-8 remained independent predictors of OS in the sunitinib arm, while no independent predictors of outcome remained in the IFN-α arm. Pharmacodynamic changes were not associated with PFS or OS for any plasma protein investigated.

**Conclusions:**

Our findings suggest that, in mRCC, baseline VEGF-A and IL-8 may have prognostic value, while baseline sVEGFR-3 may predict sunitinib efficacy.

## Introduction

The importance of angiogenesis in the growth and progression of advanced renal cell carcinoma (RCC) has been confirmed in the recent past by the success of direct or indirect antiangiogenic treatments in improving the outcome of patients with this disease [1–7]. Although prognostic criteria have been identified which enable classification of patients with advanced RCC into good, intermediate and poor risk groups [8, 9], the benefits of antiangiogenic therapy have in many cases been shown to span two or more of these groups [1, 2, 4, 6, 7]. With an increasing range of agents approved for the treatment for advanced RCC, the discovery of biological markers that reliably predict and help to monitor response to a given agent would assist clinicians in devising individual patient treatment strategies.

Sunitinib malate (SUTENT^®^) is an oral multitargeted tyrosine kinase inhibitor with potent activity against vascular endothelial growth factor receptor (VEGFR)-1, -2 and -3, platelet-derived growth factor receptor (PDGFR)-α and -β, stem cell factor receptor (KIT) and other receptor tyrosine kinases, which demonstrates antiangiogenic and antitumor activities [10–12] and is approved for the treatment of advanced RCC. In a randomized, multicenter phase III trial, sunitinib showed superior progression-free survival (PFS; the primary endpoint) to interferon-alpha (IFN-α) as first-line therapy of metastatic RCC (mRCC) with median PFS of 11 versus 5 months (*P* < 0.001) [4]. The objective response rate (ORR) was also significantly higher in the sunitinib arm (ORR 47 vs 12 %; *P* < 0.001), while median overall survival (OS) was 26.4 and 21.8 months in the sunitinib and IFN-α arms, respectively (*P* = 0.051) [4, 13].

Here, we have investigated potential biomarkers of sunitinib and IFN-α efficacy in a subset of patients enrolled in this phase III trial through the assessment of plasma levels of four soluble proteins that are closely linked to the angiogenesis process: VEGF-A, VEGF-C, a soluble extracellular fragment of VEGF receptor-3 (sVEGFR-3) and interleukin-8 (IL-8). VEGF-A is an endothelial cell-specific mitogen that is upregulated in hypoxia through stabilization of the transcription factor HIF-1α [14]; this member of the VEGF family is the primary ligand for VEGFR-2 and has been shown to mediate angiogenesis in a variety of animal models through binding to this receptor [14]. Early studies on VEGF-C and its receptor VEGFR-3 implicated these proteins in the regulation of lymph vessel formation in the adult, but more recent research indicates an additional role in mediating angiogenesis in a wide range of solid tumors [15]. VEGFR-3 is highly expressed in angiogenic sprouts in a variety of in vivo mouse models and genetic or antibody targeting of this receptor inhibits angiogenesis [16], while VEGF-C induces angiogenesis in the mouse corneal pocket assay [17]. IL-8 is a proinflammatory cytokine with proliferative and migratory activities in a variety of cell types, including tumor cells and endothelial cells [18], which stimulates angiogenesis in vivo [19, 20]. In addition to their roles in the angiogenesis process, these four proteins were selected for the present study on the basis of the results of prior correlative biomarker studies in RCC, suggesting prognostic value for circulating VEGF-A [21, 22], possible predictive or prognostic value for circulating VEGF-C and sVEGFR-3 in patients treated with sunitinib [23] and an association between elevated tumor IL-8 messenger ribonucleic acid (mRNA) expression and advanced disease [24].

## Materials and methods

### Patients

The study population comprised male and female patients aged 18 years or over with histologically confirmed mRCC with a component of clear cell histology. Key eligibility criteria included no previous systemic therapy for RCC; measurable disease; Eastern Cooperative Oncology Group (ECOG) performance status 0 or 1; and adequate hepatic, renal and cardiac function. Additional eligibility criteria have been reported previously [4]. All patients gave written informed consent.

### Study design and treatment

This phase III, multicenter study randomized 750 treatment-naïve patients with mRCC in a 1:1 ratio to receive either sunitinib in repeated 6-week cycles or IFN-α; randomization was stratified as previously described [4]. The study was run in accordance with provisions of the Declaration of Helsinki and Good Clinical Practice guidelines and was approved by the institutional review board or independent ethics committee of each participating center.

Sunitinib was administered orally at 50 mg/day for 4 weeks, followed by 2 weeks off treatment (schedule 4/2). IFN-α was administered as a subcutaneous injection on 3 non-consecutive days per week, starting at 3 million units (MU) for the first week, 6 MU for the second week and 9 MU thereafter. Treatment continued until disease progression, unacceptable toxicity or withdrawal of consent. The primary end point was progression-free survival (PFS). Tumor response and progression were determined according to the Response Evaluation Criteria in Solid Tumors (RECIST) [25], based on central radiology review of the data. Tumor assessments were performed at screening, on day 28 of cycles 1–4 and of every 2 cycles thereafter, and at the end of treatment, to confirm a response or if disease progression was suspected. An exploratory biomarker component of the study was undertaken at a limited number of participating sites.

### Assessment of biomarkers

The centers that participated in this correlative biomarker substudy were selected on a voluntary basis, driven by the scientific interest of investigators as well as the availability of suitable operational capabilities. Plasma samples were collected from all patients enrolled at the participating centers prior to dosing on days 1 and 28 of cycles 1–4 and at the end of treatment. Plasma samples were stored at −70 °C until required for analysis, and the duration of storage was within the period covered by stability evaluation. Plasma protein levels were analyzed for VEGF-A, VEGF-C, sVEGFR-3 and IL-8 using validated enzyme-linked immunosorbent assay (ELISA) kits (R&D Systems, Minneapolis, MN). The VEGF-A ELISA measures the VEGF-A165 and VEGF-A121 isoforms; the sVEGFR-3 kit measures the extracellular (soluble) domain of VEGFR-3. Assays were conducted following the manufacturer’s instructions except in the case of sVEGFR-3, for which sample dilution was 1:10 rather than 1:100 in order to increase assay sensitivity. All assays were run under Good Laboratory Practice conditions, and performance specifications of each ELISA were validated for their intended purpose per established guidelines [26].

### Statistical analysis

Soluble protein biomarker data were summarized using descriptive statistics. Assay results that were below the limit of quantitation (BLQ) and samples that were missing at time points prior to study discontinuation were excluded from the analysis. To evaluate the significance of changes in plasma protein concentrations from baseline at each time point, arithmetic differences (concentration at cycle *X* day *Y* − concentration at cycle 1 day 1) were analyzed using the Wilcoxon signed-rank test. Median PFS and OS values were estimated by the Kaplan–Meier method after stratification by the median baseline protein concentration or by the median ratio to baseline at each time point, and curves were compared using the log-rank test. Potential correlations between soluble protein values or baseline characteristics and PFS or OS were assessed by univariate and multivariate analyses using the Cox proportional hazards model and the Wald test. The baseline characteristics investigated were age; sex; number of disease sites (<3 vs ≥3); ECOG performance status (0 vs 1); nephrectomy (yes vs no); time since diagnosis (<1 vs ≥1 year); and number of risk factors (0 vs 1 or 2) as identified and published by the Memorial Sloan-Kettering Cancer Center (MSKCC; risk factors comprise low serum hemoglobin level; elevated corrected serum calcium level; elevated serum lactate dehydrogenase level; poor performance status; and interval of <1 year between diagnosis and treatment) [8]. Since the plasma proteins evaluated here were selected based on the evidence of predictive or prognostic value obtained in prior correlative studies, the present analysis evaluated specific biomarker hypotheses and adjustments for multiple comparisons were not applied.

## Results

### Comparison between biomarker subset and intent-to-treat populations

Plasma samples were collected, and protein biomarkers were measured in patients in the sunitinib (*N* = 33) and IFN-α (*N* = 30) arms who were enrolled at selected sites in this phase III study. Baseline characteristics were broadly comparable between biomarker-evaluated patients and their respective intent-to-treat (ITT) populations (Table 1), although the biomarker subset tended to have less extensive disease than the ITT population.

**Table 1:** Baseline characteristics in the biomarker subset and the intent-to-treat population, by treatment arm

	Biomarker subset (*N* = 63)		Intent-to-treat population (*N* = 750)	
	Sunitinib (*N* = 33)	IFN-α (*N* = 30)	Sunitinib (*N* = 375)	IFN-α (*N* = 375)
Gender, *n* (%)				
Male	21 (64)	24 (80)	267 (71)	269 (72)
Female	12 (36)	6 (20)	108 (29)	106 (28)
Race, *n* (%)				
White	32 (97)	25 (83)	354 (94)	340 (91)
Black	0	1 (3)	4 (1)	9 (2)
Asian	1 (3)	2 (7)	7 (2)	12 (3)
Not listed	0	2 (7)	9 (2)	13 (3)
Not allowed to ask^a^	0	0	1 (<1)	1 (<1)
Median age (range), years	64 (40–87)	62 (42–85)	62 (27–87)	59 (34–85)
ECOG performance status, *n* (%)				
0	25 (76)	20 (67)	231 (62)	229 (61)
1	8 (24)	10 (33)	144 (38)	146 (39)
Prior nephrectomy, *n* (%)	29 (88)	27 (90)	337 (90)	336 (90)
Sites of metastasis, *n* (%)				
Lung	26 (79)	24 (80)	292 (78)	297 (79)
Liver	10 (30)	12 (40)	99 (26)	90 (24)
Bone	5 (15)	7 (23)	113 (30)	112 (30)
Lymph node	20 (61)	16 (53)	218 (58)	198 (53)
Number of disease sites, *n* (%)				
1	6 (18)	9 (30)	54 (14)	73 (19)
2	12 (36)	8 (27)	107 (29)	111 (30)
≥3	15 (45)	13 (43)	214 (57)	191 (51)
Risk factors based on published MSKCC data,^b^ *n* (%)				
0 (favorable)	11 (33)	12 (41)	143 (38)	121 (34)
1–2 (intermediate)	22 (67)	17 (59)	209 (56)	212 (59)
≥3 (poor)	0	0	23 (6)	25 (7)

*ECOG* Eastern Cooperative Oncology Group, *IFN*-*α* interferon-alpha, *MSKCC* Memorial Sloan-Kettering Cancer Center

^a^By local regulation

^b^Data were missing for 17 patients in the IFN-α group (including one patient in the biomarker subset). Includes low serum hemoglobin level; elevated corrected serum calcium level; elevated serum lactate dehydrogenase level; poor performance status; and interval of <1 year between diagnosis and treatment [8]

As in the ITT population [4, 13], patients in the biomarker subset receiving sunitinib had significantly longer PFS than those receiving IFN-α [median PFS 13.7 vs 5.1 months; hazard ratio 0.293 (95 % confidence interval or CI 0.129–0.665); *P* = 0.0021]; sunitinib patients in the biomarker subset also showed a trend for improved OS compared with IFN-α patients [median OS not reached vs 18.3 months; hazard ratio 0.509 (95 % CI 0.246–1.05); *P* = 0.0626].

### Baseline plasma protein levels and ratios to baseline on treatment with sunitinib or interferon-α

None of the assay results for VEGF-A or sVEGFR-3 were BLQ, while the proportion of assay results for VEGF-C and IL-8 that were BLQ was 0.52 and 0.78 %, respectively. Median baseline plasma VEGF-A concentrations in both the sunitinib arm (49.2 pg/mL, *N* = 33) and the IFN-α arm (53.1 pg/mL, *N* = 30) were significantly higher than median VEGF-A levels in healthy volunteers (14.8 pg/mL, *N* = 15; *P* < 0.0001 in each case). Similarly, median baseline plasma VEGF-C concentrations in both the sunitinib arm (493.7 pg/mL, *N* = 33) and the IFN-α arm (503.3 pg/mL, *N* = 30) were significantly higher than median VEGF-C levels in healthy volunteers (207.6 pg/mL, *N* = 19; *P* < 0.0001 in each case). Median baseline plasma sVEGFR-3 concentrations in the sunitinib arm (38.9 ng/mL, *N* = 33) and the IFN-α arm (37.6 ng/mL, *N* = 30), and median baseline IL-8 concentrations in the sunitinib arm (7.0 pg/mL, *N* = 31) and the IFN-α arm (9.5 pg/mL, *N* = 29) did not differ significantly from median baseline levels of sVEGFR-3 (43.6 ng/mL, *N* = 20) and IL-8 (6.5 pg/mL, *N* = 20) in healthy volunteers. In no case did baseline plasma protein concentrations in the sunitinib arm differ significantly from those in the IFN-α arm.

**Fig. 1:**
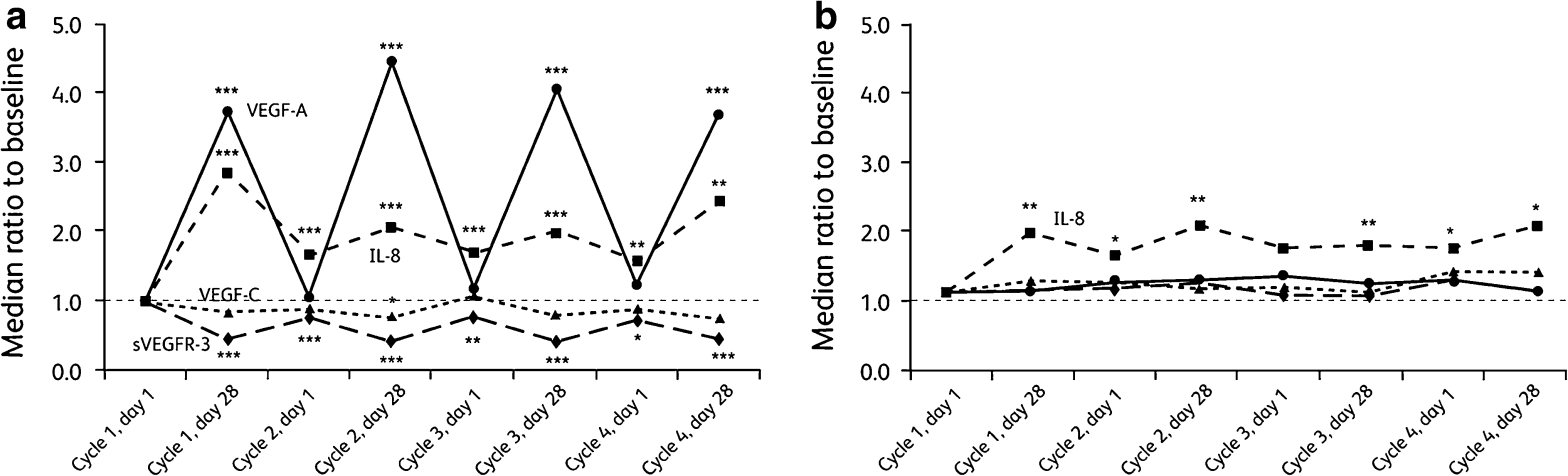
Change in biomarker [vascular endothelial growth factor (VEGF)-A, VEGF-C, interleukin-8 (IL-8) and soluble vascular endothelial growth factor receptor-3 (sVEGFR-3)] levels during treatment in the **a** sunitinib arm and **b** interferon-alpha arm. *P < 0.05; **P < 0.01; ***P < 0.0001 compared with baseline

In the sunitinib arm, median plasma levels of VEGF-A increased reversibly compared with baseline by more than fourfold during treatment, while those of IL-8 increased by approximately twofold to threefold on treatment and showed less reversibility than VEGF-A after 2-week off-drug periods (Fig. 1); median increases from baseline levels were significant in all cases. Changes in median plasma VEGF-C levels during treatment were minimal, while median sVEGFR-3 levels decreased reversibly and significantly by approximately 50 %. In the IFN-α arm, plasma IL-8 levels were significantly elevated approximately twofold above baseline on study, while levels of the other proteins did not change (Fig. 1).

### Relationship between soluble protein levels and efficacy end points

**Fig. 2:**
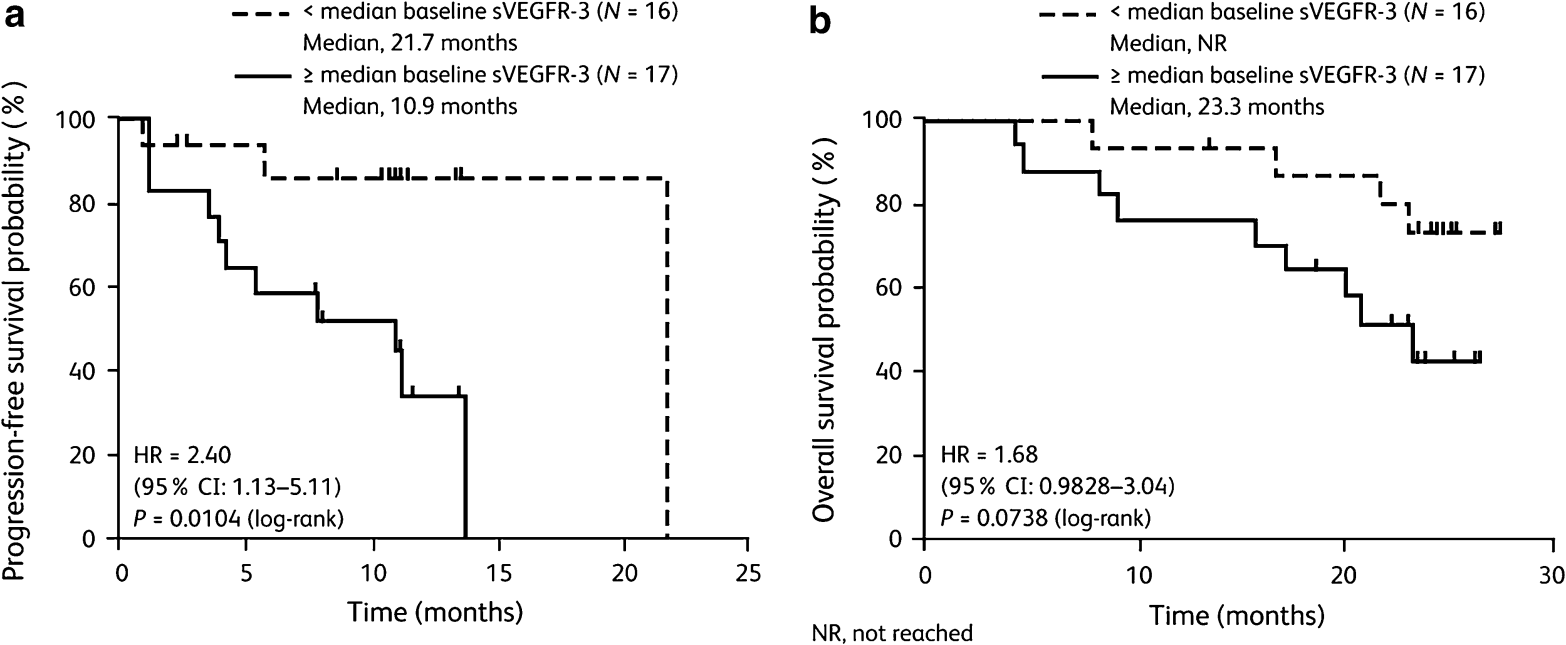
Sunitinib arm: **a** progression-free and **b** overall survival after stratification by median baseline plasma soluble vascular endothelial growth factor receptor-3 (sVEGFR-3)

Comparison of Kaplan–Meier time-to-event curves after patient stratification by median baseline soluble protein concentrations showed that, in the sunitinib arm, patients with baseline VEGF-A levels below the median had significantly longer PFS than those with baseline VEGF-A greater than or equal to the median (median PFS 13.7 vs 7.8 months; *P* = 0.0059; Table 2); OS was also significantly improved in patients with baseline VEGF-A below the median (median OS not reached vs 21.8 months; *P* = 0.0043; Table 2). Similar results were obtained for baseline sVEGFR-3 in sunitinib-treated patients (Fig. 2), although the difference in OS did not reach significance (Table 2). In the IFN-α arm, patients with baseline IL-8 below median levels had significantly longer PFS and a trend for longer OS than those with baseline IL-8 greater than or equal to the median (median PFS 7.8 vs 2.6 months; *P* = 0.0472; Table 2). When PFS was compared between the sunitinib and IFN-α arms in patient subsets having relatively low (below the median) or relatively high (greater than or equal to the median) baseline levels of each soluble protein, hazard ratios favored sunitinib in all analyses. In each case, the treatment effect was greater for patients with relatively low soluble protein levels at baseline (Table 3). There were no significant associations between pharmacodynamic changes in the soluble proteins investigated and either PFS or OS (data not shown).

**Table 2:** Within-treatment comparison of progression-free and overall survival stratified by median baseline soluble protein concentrations

Soluble protein	Treatment arm^a^	Median PFS (months)		HR (95 % CI)	Log-rank *P*
		<Median [protein]	≥Median [protein]		
VEGF-A	Sunitinib	13.7	7.8	2.55 (1.19–5.48)	0.0059
	IFN-α	7.8	3.9	1.35 (0.805–2.27)	0.248
VEGF-C	Sunitinib	13.7	11.1	1.42 (0.779–2.59)	0.242
	IFN-α	5.1	7.8	1.12 (0.619–2.01)	0.713
sVEGFR-3	Sunitinib	21.7	10.9	2.40 (1.13–5.11)	0.0104
	IFN-α	5.4	3.9	1.16 (0.688–1.95)	0.582
IL-8	Sunitinib	21.7	12.4	1.20 (0.685–2.1)	0.524
	IFN-α	7.8	2.6	1.84 (1.055–3.47)	0.0472

Plasma protein	Treatment arm^a^	Median OS (months)		HR (95 % CI)	Log-rank *P*
		<Median [protein]	≥Median [protein]		
VEGF-A	Sunitinib	NR	21.8	2.60 (1.22–5.53)	0.0043
	IFN-α	26.6	12.8	1.49 (0.903–2.45)	0.11
VEGF-C	Sunitinib	NR	23.3	1.51 (0.837–2.72)	0.159
	IFN-α	22.0	16.5	1.01 (0.619–1.66)	0.961
sVEGFR-3	Sunitinib	NR	23.3	1.68 (0.928–3.04)	0.0738
	IFN-α	22.0	17.6	1.24 (0.757–2.04)	0.388
IL-8	Sunitinib	NR	23.3	1.37 (0.761–2.48)	0.283
	IFN-α	22.0	12.9	1.52 (0.924–2.51)	0.0897

*CI* confidence interval, *HR* hazard ratio, *IL*-*8* interleukin-8, *IFN*-*α* interferon-alpha, *NR* not reached, *PFS* progression-free survival, *OS* overall survival, *VEGF*-*A* vascular endothelial growth factor A, *VEGF*-*C* vascular endothelial growth factor C, *sVEGFR*-*3* soluble vascular endothelial growth factor receptor 3

^a^Total *N* = 33 and *N* = 30 for each protein in the sunitinib and IFN-α arms, respectively, apart from IL-8, where total *N* = 31 and *N* = 29 in the sunitinib and IFN-α arms, respectively

**Table 3:** Comparison of progression-free survival between sunitinib and interferon-alpha arms in patient subsets having relatively low (<median) or high (≥median) baseline levels of each soluble protein

Patient subset^a^	Median PFS (months)		HR (95 % CI)	Log-rank *P*
	Sunitinib	IFN-α		
VEGF-A				
<Median	13.7	7.8	0.148 (0.038–0.579)	0.0019
≥Median	10.9	3.9	0.451 (0.149–1.36)	0.150
VEGF-C				
<Median	13.7	5.1	0.190 (0.059–0.617)	0.0022
≥Median	11.1	7.8	0.551 (0.156–1.94)	0.348
sVEGFR-3				
<Median	21.7	5.4	0.122 (0.024–0.615)	0.0034
≥Median	10.9	3.9	0.406 (0.147–1.12)	0.0719
IL-8				
<Median	21.7	7.8	0.247 (0.077–0.787)	0.0114
≥Median	13.7	2.6	0.403 (0.12–1.35)	0.131

*CI* confidence interval, *HR* hazard ratio, *IL*-*8* interleukin-8, *IFN*-*α* interferon-alpha, *PFS* progression-free survival, *VEGF*-*A* vascular endothelial growth factor A, *VEGF*-*C* vascular endothelial growth factor C, *sVEGFR*-*3* soluble vascular endothelial growth factor receptor 3

^a^Total *N* = 33 and *N* = 30 for each protein in the sunitinib and IFN-α arms, respectively, apart from IL-8, where total *N* = 31 and *N* = 29 in the sunitinib and IFN-α arms, respectively

Baseline plasma protein concentrations and ratio to baseline values (expressed as continuous variables) and baseline clinical characteristics were also analyzed using the Cox proportional hazards model. For each soluble protein analyzed, low plasma concentrations at baseline were associated with a lower risk of progression and death (Table 4). In univariate analysis for both treatment arms, baseline VEGF-A, IL-8 and prior nephrectomy correlated with OS while baseline VEGF-C correlated with PFS (Table 4). In the sunitinib arm, baseline VEGF-A correlated with PFS, and baseline sVEGFR-3 correlated with both PFS and OS. In the IFN-α arm, baseline IL-8 and prior nephrectomy also correlated with PFS, while number of disease sites correlated with OS (Table 4). In multivariate analysis, only baseline sVEGFR-3 and baseline IL-8 were significant predictors of OS in the sunitinib arm, while none were significant in the IFN-α arm (Table 4).

**Table 4:** Univariate and multivariate analyses of progression-free survival and overall survival by treatment arm using the Cox proportional hazards model

Variables [*n* for each group]	PFS		OS	
	HR (95 % CI)	Wald *P*	HR (95 % CI)	Wald *P*
Sunitinib				
Univariate analysis				
Age (<65 vs ≥65 years) [17, 16]	0.595 (0.179–1.99)	0.3990	0.698 (0.228–2.14)	0.5290
Sex (M vs F) [21, 12]	0.893 (0.267–2.99)	0.8540	0.779 (0.24–2.53)	0.6780
No. of disease sites (<3 vs ≥3) [18, 15]	1.16 (0.383–3.47)	0.7950	2.12 (0.694–6.51)	0.1870
ECOG performance status (0 vs 1) [25, 8]	1.58 (0.474–5.26)	0.4540	2.37 (0.773–7.25)	0.1310
Nephrectomy (no vs yes) [4, 29]	0.269 (0.071–1.02)	0.0531	0.197 (0.053–0.729)	0.0150
Time since diagnosis (<1 vs ≥1 year) [21, 12]	0.301 (0.066–1.37)	0.1210	0.274 (0.061–1.24)	0.0921
Risk factors based on published MSKCC data^a^ (0 vs 1 or 2) [11, 22]	2.74 (0.599–12.5)	0.1940	6.84 (0.888–52.6)	0.0649
Baseline VEGF-A (ng/10 mL) [33]	1.28 (1.06–1.55)	0.0108	1.33 (1.09–1.63)	0.0052
Baseline VEGF-C (ng/mL) [33]	6.42 (1.25–33)	0.0259	2.91 (0.703–12.1)	0.1410
Baseline sVEGFR-3 (ng/mL) [33]	1.04 (1.01–1.06)	0.0026	1.04 (1.02–1.07)	0.0012
Baseline IL-8 (pg/mL) [31]	1.05 (0.991–1.11)	0.1010	1.08 (1.03–1.14)	0.0026
Multivariate analysis				
Nephrectomy (no vs yes) [4, 29]	–	–	0.385 (0.065–2.28)	0.290
Baseline VEGF-A (ng/10 mL) [33]	1.05 (0.677–1.63)	0.82	0.559 (0.310–1.01)	0.053
Baseline VEGF-C (ng/mL) [33]	4.84 (0.665–35.23)	0.12	–	–
Baseline sVEGFR-3 (ng/mL) [33]	1.03 (0.975–1.09)	0.300	1.064 (1.004–1.13)	0.037
Baseline IL-8 (pg/mL) [31]	–	–	1.110 (1.022–1.20)	0.013
Interferon-alpha				
Univariate analysis				
Age (<65 vs ≥65 years) [16, 14]	3.87 (0.967–15.5)	0.0558	1.21 (0.454–3.23)	0.7040
Sex (M vs F) [24, 6]	1.36 (0.347–6.89)	0.7220	1.92 (0.612–6.02)	0.2640
No. of disease sites (<3 vs ≥3) [17, 13]	1.16 (0.408–3.31)	0.7780	3.78 (1.39–10.3)	0.0092
ECOG performance status (0 vs 1) [20, 10]	1.88 (0.656–1.39)	0.2400	2.05 (0.735–5.7)	0.1700
Nephrectomy (no vs yes) [3, 27]	0.06 (0.008–0.418)	0.0049	0.09 (0.018–0.478)	0.0044
Time since diagnosis (<1 vs ≥1 year) [14, 16]	1.18 (0.404–3.44)	0.7630	0.68 (0.252–1.83)	0.4450
Risk factors based on published MSKCC data^a^ (0 vs 1 or 2) [12, 17]	0.77 (0.268–2.22)	0.6310	1.99 (0.631–6.27)	0.2410
Baseline VEGF-A (ng/10 mL) [30]	3.05 (0.48–19.4)	0.237	1.24 (1.04–1.47)	0.0174
Baseline VEGF-C (ng/mL) [30]	3.64 (1.22–10.9)	0.0206	2.12 (0.917–4.89)	0.0790
Baseline sVEGFR-3 (ng/mL) [30]	1.01 (0.971–1.06)	0.5270	1.04 (1–1.08)	0.0505
Baseline IL-8 (pg/mL) [29]	1.04 (1–1.07)	0.0423	1.02 (1–1.03)	0.0094
Multivariate analysis				
No. of disease sites (<3 vs ≥3) [17, 13]	–	–	2.428 (0.752–7.84)	0.140
Nephrectomy (no vs yes) [3, 27]	0.113 (0.008–1.52)	0.100	0.218 (0.028–1.67)	0.140
Baseline VEGF-A (ng/10 mL) [30]	–	–	0.925 (0.525–1.63)	0.790
Baseline VEGF-C (ng/mL) [30]	1.470 (0.158–13.6)	0.740	–	–
Baseline IL-8 (pg/mL) [29]	1.018 (0.964–1.08)	0.520	1.013 (0.969–1.06)	0.560

For binary variables, a hazard ratio <1 represents risk reduction for the first category and a hazard ratio >1 represents risk reduction for the second category; for continuous variables (i.e., the soluble proteins), a hazard ratio >1 equates to risk reduction when the value decreases and a hazard ratio <1 equates to risk reduction when the value increases

Variables that were significant (*P* < 0.05) by univariate analysis were evaluated in the multivariate model

*CI* confidence interval, *ECOG* Eastern Cooperative Oncology Group, *HR* hazard ratio, *MSKCC* Memorial Sloan-Kettering Cancer Center, *PFS* progression-free survival, *OS* overall survival

^a^Includes low serum hemoglobin level; elevated corrected serum calcium level; elevated serum lactate dehydrogenase level; poor performance status; and interval of <1 year between diagnosis and treatment [8]

## Discussion

In this study, we have investigated the plasma pharmacodynamics of a panel of circulating proteins linked to the mechanism of action of sunitinib (VEGF-A, VEGF-C, sVEGFR-3), as well as IL-8, a potential mediator of resistance to VEGFR inhibition, in a subset of patients in a randomized phase III study comparing sunitinib and IFN-α as first-line treatment for mRCC. In addition, we have explored possible associations between baseline levels of these proteins, or changes from baseline at each time point, and clinical outcome. Significant and consistent changes from baseline levels were seen for plasma VEGF-A, IL-8 and sVEGFR-3 in the sunitinib arm and for plasma IL-8 in the IFN-α arm. In both treatment arms, baseline levels of plasma VEGF-A, VEGF-C and IL-8 were significantly associated with PFS or OS, while baseline plasma sVEGFR-3 was significantly associated with PFS and OS in the sunitinib arm only. No significant and consistent correlations were seen between plasma protein changes from baseline and clinical outcome in either treatment arm.

Our findings provide additional support for the hypothesis that circulating VEGF-A is prognostic for OS in RCC, with low baseline concentrations of VEGF-A correlating with longer OS in both sunitinib and IFN-α arms in the present study. Consistent with these results, Peña et al. [21] observed that low baseline serum VEGF-A levels in the placebo arm correlated with longer OS by univariate analysis in a placebo-controlled phase III study of sorafenib, a multitargeted kinase inhibitor with potent activity against the 3 VEGF receptors. As observed here, significance was not seen by multivariate analysis when other circulating biomarkers were included. Also, no correlations were observed between the change in biomarker levels (from baseline to week 3 or 12) and outcome (PFS or OS) in sorafenib-treated patients. With respect to PFS in sunitinib-treated RCC patients, our finding of an association between low baseline VEGF-A and prolonged PFS is similar to that reported by Porta et al. [27] in a biomarker study from the sunitinib expanded access program; assessment of potential correlations with OS was not included in that study. Although Rini et al. [23] did not observe a correlation between baseline VEGF-A levels and PFS in a phase II study of sunitinib in mRCC patients refractory to the anti-VEGF-A antibody bevacizumab, this negative result may have been influenced by prior VEGF-A pathway inhibition by bevacizumab in this patient population, or by the confounding effects of residual bevacizumab in plasma samples on antibody-based VEGF-A measurement by ELISA. In that phase II sunitinib study, baseline VEGF-A concentrations were inversely correlated with time since final bevacizumab treatment. Other studies in RCC patients have linked high baseline VEGF-A concentrations with shorter survival time, higher clinical stage and higher tumor grade [28–30] and, in some cases, have identified baseline VEGF-A as an independent prognostic factor for PFS and/or OS [22, 31].

The phase II sunitinib RCC study cited earlier that involved patients refractory to bevacizumab [23] demonstrated an association between low baseline sVEGFR-3 concentrations and prolonged PFS, consistent with the association reported here for the sunitinib arm. The absence of a significant association between sVEGFR-3 and either PFS or OS in the IFN-α arm of the present study, although trending toward significance for OS, suggests that sVEGFR-3 may be more predictive of sunitinib efficacy than prognostic in RCC, but more research is necessary to address this question. Early reports implicated VEGFR-3 exclusively in the process of lymph vessel production (lymphangiogenesis), but more recent studies have shown that this receptor for VEGF-C and VEGF-D (but not VEGF-A) is expressed both in tumor lymph vessels and in tumor endothelium in a variety of malignancies [32–34]. Although lymphangiogenic activity appears to be relatively low in clear cell RCC [35, 36], our correlative findings for sVEGFR-3 suggest a possible role for the inhibition of lymphangiogenesis in the clinical activity of sunitinib in this disease, in addition to antiangiogenesis. Further research is required to distinguish between these possibilities. Also of interest is the recent paper by Garcia-Donas et al. [37], which reported strong associations between two VEGFR3 polymorphisms and PFS in RCC patients treated with sunitinib. Taken together, these findings strongly implicate VEGFR-3 as a potential target for sunitinib that may contribute to efficacy in patients with mRCC.

IL-8 (CXCL8) is a CXC family chemokine that activates multiple signaling pathways, increases proliferation and survival of both endothelial and tumor cells and facilitates the migration of these cell types [18]. In addition, IL-8 possesses potent proangiogenic activity in vivo [19, 20]. Tumor IL-8 expression is upregulated in RCC and has been associated both with more advanced disease and with poor survival [24, 38, 39]. Consistent with these published reports, the present findings suggest that high baseline plasma levels of IL-8 are associated with poor prognosis in RCC; plasma IL-8 concentrations correlated inversely with OS in both treatment arms and correlated inversely with PFS in the IFN-α arm, remaining an independent predictor of OS in the sunitinib arm. Studies with the potent VEGF receptor inhibitor pazopanib have also shown that IL-8 is prognostic for outcome in RCC, as well as predictive of response [40, 41]. Preclinically, IL-8 has recently been shown to mediate sunitinib resistance in animal models of RCC [42]. These investigators found that sunitinib-resistant renal tumors were more highly vascularized than those that were sensitive and hypothesized that the tumors had escaped from the antiangiogenic effects of sunitinib by activation of a VEGF/VEGFR-independent mechanism. Screening of xenograft-bearing mice for changes in plasma levels of 89 angiogenic factors revealed that human IL-8 levels were significantly elevated in sunitinib-resistant mice, while levels of human VEGF-A (and other factors) were unchanged. Furthermore, neutralization of IL-8 activity partially restored sunitinib sensitivity in these preclinical models. In a small prospective study, the same authors also found that baseline IL-8 expression was significantly higher in tumor specimens from RCC patients with intrinsic resistance to sunitinib than in tumor specimens from patients who did not progress on treatment. Overall, preclinical and clinical investigations have provided in vivo evidence of a role for IL-8 as a mediator of tumor progression in RCC and as a possible mechanism of innate or acquired resistance to antiangiogenic therapy in this disease.

Plasma IL-8 levels were significantly elevated above baseline at all time points in both arms of this randomized study. Hypoxia has been shown to upregulate IL-8 expression in human rhabdomyosarcoma cell lines, in a manner that is independent of HIF-1α activity [43]. A similar mechanism might account for plasma IL-8 induction in the present study, even in the presence of VEGF receptor blockade in sunitinib-treated patients. Although hypoxia also induces IL-8 expression in cultured endothelial cells [44], this response is mediated by VEGF-A and is therefore unlikely to account for elevated plasma IL-8 levels following sunitinib treatment. In vivo evidence for hypoxia-induced IL-8 expression was obtained in D-12 melanoma xenografts, in which IL-8 expression was seen in vascular hot spots that were associated with hypoxic foci [45]. In support of a role for hypoxia in IL-8 induction in the IFN-α arm, IFN-α has been shown to possess antiangiogenic activity [46, 47], in addition to its immunomodulatory properties. However, the finding that plasma VEGF-A levels did not significantly change in response to IFN-α treatment, whereas a marked and significant increase in VEGF-A from baseline levels was seen at the end of each treatment period in the sunitinib arm, suggests that a mechanism other than hypoxia induction may be involved in plasma IL-8 induction in IFN-α-treated patients. Clearly, further research is required to explore possible mechanism(s) underlying plasma IL-8 pharmacodynamics in the present study.

Accumulating data suggests an influence of germline polymorphisms on RCC patient efficacy and safety when receiving targeted anti-VEGF or VEGFR2 tyrosine kinase inhibitor therapies. Specifically, publications have cited polymorphisms in the VEGF-A gene [48] or VEGFR-3 as associating with clinical outcome [49]. However, the absence of concordance of findings, different SNPs assayed and endpoints measured preclude use of germline polymorphism data for patient selection at the current time. Large multicenter prospective RCC studies in which baseline characteristics, clinical outcomes and SNPs are measured will allow for unequivocal assignment of utility of SNPs for patient selection.

A number of limitations apply to the present investigation. Firstly, only selected sites participated in the exploratory biomarker component of this pivotal phase III study. As a result, samples sizes in the sunitinib and IFN-α arms (33 and 30, respectively, at baseline) were small in relation to patient numbers in the corresponding ITT populations (less than 10 % in each case). The power to detect significant correlations between plasma proteins and clinical outcome was thus markedly reduced, and the possibility of type II errors was markedly increased, when compared with a biomarker analysis involving all patients on study. Nonetheless, a number of significant associations with PFS or OS were observed for soluble proteins in pretreatment plasma samples, and it should be noted that baseline characteristics and clinical outcome (PFS, OS) for the biomarker-evaluated patient subsets in the two treatment arms were a good representation of their respective full study populations. Another limitation of this study is that we have focused on a small panel of circulating proteins, yet there are many proteins not directly linked to sunitinib’s molecular mechanism, of which IL-8 is but one example, that have the potential to predict resistance to sunitinib therapy in RCC based on a known regulatory role in angiogenesis. Circulating biomarkers, including plasma proteins, have the distinct practical advantage of relatively facile sampling and quantitative analysis when compared with tumor tissue-based end points. However, the search for circulating proteins with utility in clinical decision-making for any approved agent in RCC, or indeed any other tumor type, has proven largely unsuccessful, likely in part because the data collected often reflect systemic processes to a greater extent than the relevant tumor biology. Finally, we have interpreted significant associations that were obtained for OS in both treatment arms to suggest prognostic rather than predictive value for a biomarker. However, since the efficacy of IFN-α in RCC may be mediated in part by angiogenesis inhibition, prediction of survival for such a biomarker may not be entirely independent of treatment modality.

In summary, the present investigation provides evidence that plasma concentrations of VEGF-A and IL-8 may be prognostic for OS in mRCC, with high levels being unfavorable. In addition, low plasma levels of the soluble form of VEGFR-3 may predict improved outcome for RCC patients receiving sunitinib, suggesting that inhibition of angiogenesis and/or lymphangiogenesis mediated by this VEGF receptor family member may contribute to the efficacy of this potent multitargeted tyrosine kinase inhibitor. Further predictive biomarker research is clearly warranted in mRCC, not only for sunitinib but also for other VEGF pathway inhibitors and for agents targeting other pathways.
